# Disruption of *Lrpprc* affects B cell development and proliferation in a mouse model of Leigh Syndrome French Canadian type

**DOI:** 10.1007/s44162-025-00094-x

**Published:** 2025-07-01

**Authors:** Adrien Fois, Sonia Deschênes, Capucine Bourel, Claudine Beauchamp, Félix Lombard-Vadnais, Matthieu Ruiz, Guy Charron, Lise Coderre, LSFC Consortium, Christine Des Rosiers, Azadeh Alikashani, Bruce G. Allen, Chantale Aubut, Chantal Bémeur, Yan Burelle, François Labarthe, Jeannine Landry, Catherine Laprise, Geneviève Lavallée, Pierre Lavoie, Bruno Maranda, Charles Morin, Yvette Mukaneza, Tamiko Nishimura, Marie-Ève Rivard, Florin Sasarman, Eric A. Shoubridge, Jessica Tardif, Julie Thompson Legault, Nancy Tremblay, Vanessa Tremblay-Vaillancourt, Luc Vachon, Josée Villeneuve, John D. Rioux, Sylvie Lesage

**Affiliations:** 1 Axe Immunologie-Oncologie, Centre de Recherche de L’Hôpital Maisonneuve-Rosemont, Montréal, Québec Canada; 2 Département de Microbiologie, infectiologie et immunologie, Université de Montréal, Montréal, Québec Canada; 3 Centre de Recherche, Institut de Cardiologie de Montréal, Montréal, Québec Canada; 4 Département de Nutrition, Université de Montréal, Montréal, Québec Canada; 5 Département de Médecine, Université de Montréal, Montréal, Québec Canada

**Keywords:** LSFC, *LRPPRC*, Immune cells, Mitochondria, Proliferation, B cells

## Abstract

**Purpose:**

Leigh Syndrome French Canadian (LSFC) is a rare autosomal recessive metabolic disorder characterized by severe lactic acidosis crises and early mortality. LSFC patients carry variants in the Leucine Rich Pentatricopeptide Repeat Containing (*LRPPRC*) nuclear gene, which lead to defects in the respiratory chain complexes and mitochondrial dysfunction. Mitochondrial respiration modulates cellular metabolic activity, which impacts many cell processes, including the differentiation and function of immune cells. The purpose of this study is to define the role of *Lrpprc* on immune cell function.

**Methods:**

As genetic deletion of *Lrpprc* is not viable, we generated two conditional mouse models: a model for systemic deletion of *Lrpprc* and a knock-in (KI) model carrying the most common LSFC pathogenic variant in Quebec, NM_133259.4(LRPPRC):c.1061C > T (p.Ala354Val).

**Results:**

We demonstrate that *Lrpprc* is an essential gene even in adult mice, as systemic deletion of *Lrpprc* leads to prominent weight loss and mortality. We also find an increase in lactate levels, a symptom of metabolic crises in LSFC. *Lrpprc* deletion and pathogenic variant affect various immune cell subsets, with a strong impact on B cell development and proliferation.

**Conclusions:**

We generated a viable disease-relevant mouse model to study the role of *Lrpprc* in vivo and find that disruption of *Lrpprc* strongly impairs B cell development and proliferation.

**Supplementary Information:**

The online version contains supplementary material available at 10.1007/s44162-025-00094-x.

## Introduction

Leigh syndrome is a rare and devastating neurodegenerative disease that causes severe neurological impairment and cognitive decline [1, 2]. A variant of this disease has been described in the Saguenay-Lac-Saint-Jean region of Quebec (Canada), called Leigh Syndrome French Canadian (LSFC) [3, 4]. In addition to cognitive decline, people living with LSFC suffer from unpredictable and often lethal acute metabolic acidosis crises, with a median age of death at 1.6 years old in > 80% of individuals [4, 5]. Despite high death rate and carrier screening [6], new cases continue to arise. For the survivors, each crisis results in a significant deterioration of health status [3–5]. Initially, two *LRPPRC* pathogenic variants were identified as causative of LSFC. The most common homozygous substitution is in exon 9, namely NM_133259.4(LRPPRC):c.1061 C > T (p.Ala354Val), hereafter denoted as Ala354Val [7]. The second variant identified in Quebec, NM_133259.4(LRPPRC):c.3830_3837 del, p.(Cys1277*), is in exon 35. The individual carrying this variant is a compound heterozygote with the Ala354Val variant [7]. More recently, new pathogenic variants have been identified outside the Quebec population in distinct families from England, Turkey, Iraq, India, China, Saudi Arabia and Italy [8–12]. Even with the identification of new *LRPPRC* variants causing LSFC, this syndrome remains extremely rare. Notwithstanding the early mortality, there are approximately ten individuals living with LSFC in Quebec, whereas the new variants outside Quebec have been identified in a few individuals in specific families.

*LRPPRC* is a nuclear gene and the protein primarily localizes to the mitochondria, where it stabilizes mitochondrial mRNAs [7, 13–15]. The mitochondria is a key organelle in cellular metabolism, particularly for the oxidative phosphorylation carried out by the complexes in the respiratory chain [16]. Metabolism plays a crucial role in the development and function of immune cells, with each immune cell type exhibiting specific metabolic preferences [17–19]. Reduced expression of LRPPRC, due to the Ala354Val variant [20], leads to disruption of the mitochondrial respiratory chain, particularly complex IV (cytochrome c oxidase) [21–23]. Consequently, variants in *LRPPRC* may induce alterations in the immune system, increasing susceptibility to infections in people living with LSFC [24]. In line with this observation, we previously demonstrated that LSFC patients display impaired humoral response to the measles, mumps and rubella (MMR) vaccine [25].

To understand the role of *LRPPRC* in the immune system and the impact of relevant LSFC variants, we generated conditional knock-out and knock-in mouse models that recapitulate key features of LSFC. We found that *Lrpprc* deficiency induces important changes in the composition of the immune system, especially at the level of B cell development, by inhibiting cellular proliferation.

## Results

### Validation of LSFC mouse models

To study the impact of *LRPPRC* variant and disruption on immune cells, we generated *Lrpprc*^Ala354Val^ mice, a mouse model carrying the Ala354Val orthologous variant identified in people living with LSFC. Unfortunately, as for full body *Lrpprc* knock-out (KO) mice, mice homozygous for the variant (*Lrpprc*^Ala354Val/Ala354Val^, hereafter *Lrpprc*^KI/KI^) are not viable (data not shown). We therefore took advantage of the *Lrpprc*^fl/fl^ mouse [26], to create two novel conditional knock-out mouse models, namely the *Lrpprc*^fl/fl^.GT-Rosa^cre/ERT2+/−^ and the *Lrpprc*^fl/KI^.GT-Rosa^cre/ERT2+/−^ mice, which respectively carry the *Lrpprc*^−/−^ and *Lrpprc*^−/KI^ genotypes after tamoxifen administration. These mouse models allow us to study the impact of *Lrpprc* deletion as well as the impact of the most common pathogenic variant, namely Ala354Val.

**Fig. 1:**
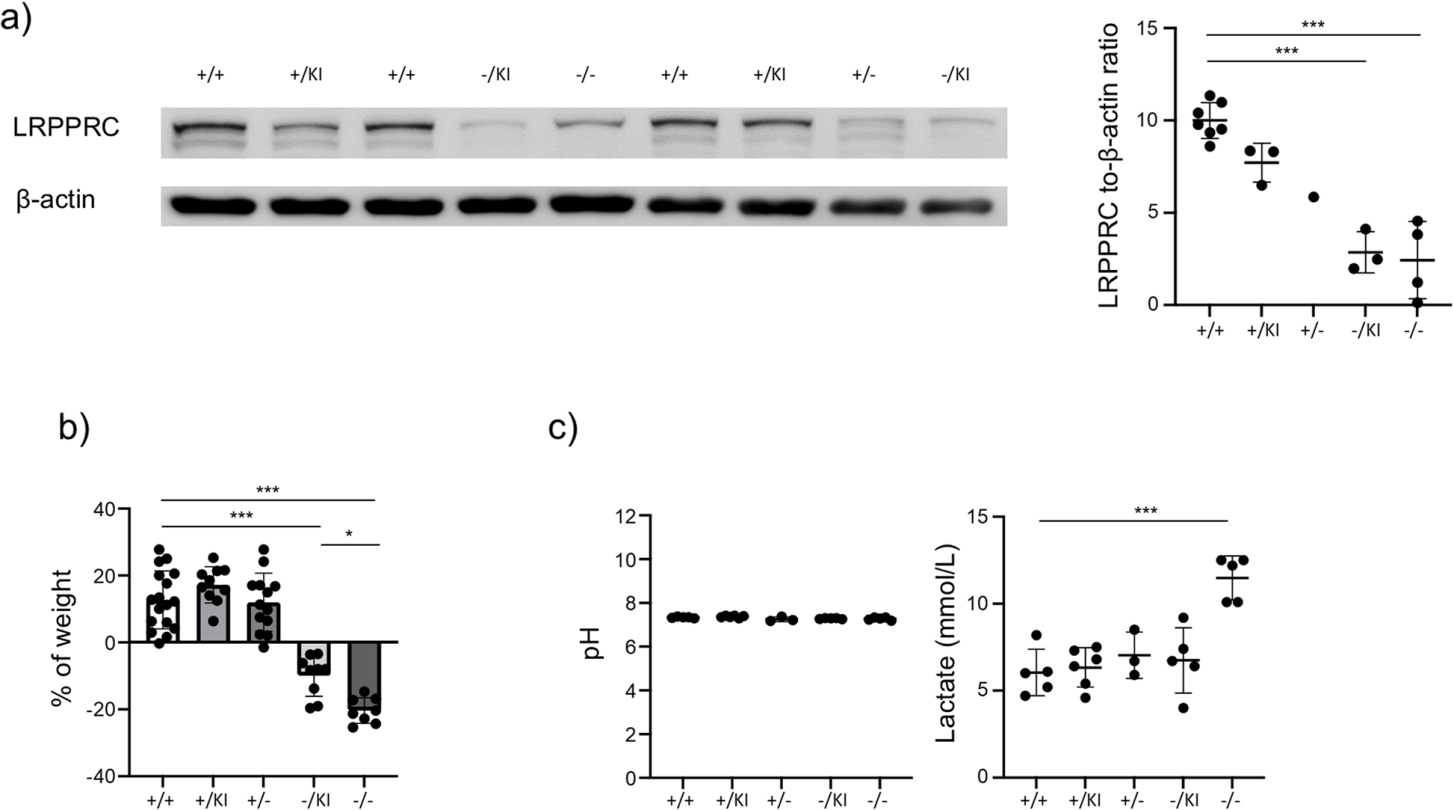
Validation of LSFC mouse models. **a** Left, immunoblotting analysis of Lrpprc from spleen cells from mice six weeks after gavage. The top band corresponds to the molecular weight of Lrpprc. Representative images of three independent experiments. Right, compilations of Lrpprc on β-actin ratio (*n* = 1–7). **b** Weight evolutions of mice six weeks after gavage compared to the day of the first gavage (*n* = 8–17). **c** Blood parameters of mice six weeks after gavage. The data were acquired from at least three independent experiments. Each dot represents data from an individual mouse and the dash depicts the mean with the standard deviation. *P < 0.05; *** *P* < 0.001. The post-gavage *Lrpprc* genotypes are indicated on the x axes

We subjected adult mice to tamoxifen gavage, waited six weeks, and quantified the level of Lrpprc expression by western blot. We find that Lrpprc shows a graded decrease in expression level based on genotype, reaching a 75% decrease in *Lrpprc*^−/KI^ and *Lrpprc*^−/−^ mice relative to control (Fig. 1a). Six weeks after gavage, while control mice and mice expressing at least one WT *Lrpprc* allele gained ~ 15% in body weight, *Lrpprc*^**−/**KI^ mice lost ~ 10% of their initial weight (Fig. 1b). *Lrpprc*^−/−^ mice showed an even more drastic weight loss, with ~ 20% weight loss six weeks post-gavage (Fig. 1b), at which point mice had to be euthanatized. This suggests that *Lrpprc* is an essential gene even in adult mice.

An important clinical manifestation of LSFC is the onset of metabolic acidosis crises, characterized by a decrease in pH and an increase in blood lactate levels [3, 5]. We thus quantified the pH and blood lactate levels in the various mice, six weeks after gavage. Although the pH remained unchanged, we observed an increase in blood lactate in *Lrpprc*^−/−^ mice (Fig. 1c), an early sign of metabolic acidosis. The increase in lactate was not observed in *Lrpprc*^**−/**KI^, suggesting that the presence of the Ala354Val mutated protein is sufficient to maintain lactate homeostasis when the mice are not subjected to stresses. Taken together, these results suggest that disruption of *Lrpprc* in mice replicates at least some clinical observations of people living with LSFC.

### Lrpprc *disruption impacts the lymphocyte pool*

**Fig. 2:**
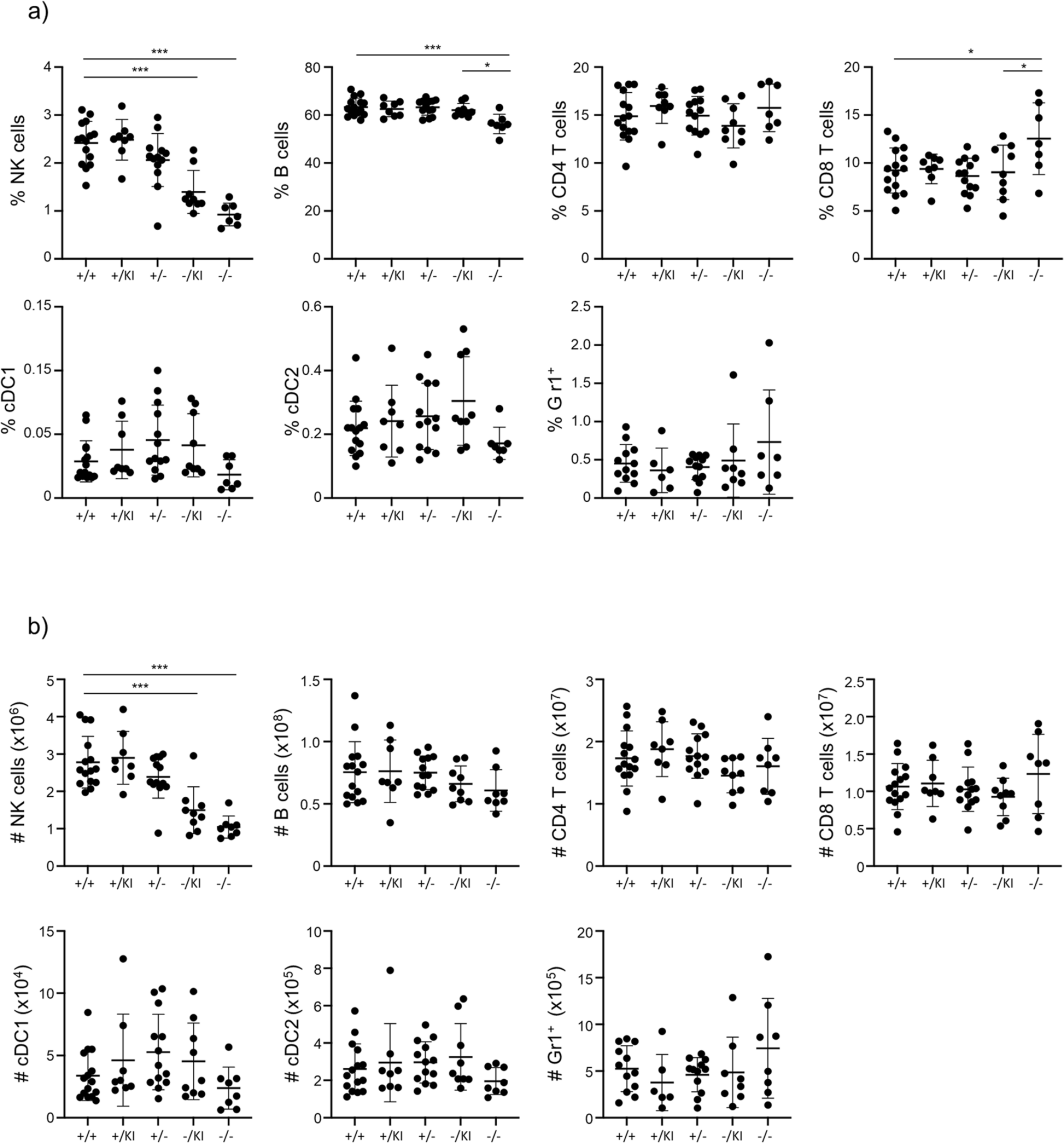
*Lrpprc* disruption impacts the lymphocyte pool. **a** Compilation of the percentage (*n* = 7–15) and **b** absolute number (*n* = 8–15) of spleen cells gated as detailed in supplementary Fig. 1a. The data were acquired from at least three independent experiments. Each dot represents data from an individual mouse, and the dash depicts the mean with the standard deviation. *P < 0.05; *** *P* < 0.001. The post-gavage *Lrpprc* genotypes are indicated on the x axes

We have previously investigated the response of LSFC patients to the MMR vaccine and observed an impaired humoral response and reduced protection for some patients [25]. This observation led us to investigate the impact of *Lrpprc* on the immune system in our mouse models. First, we performed general immune phenotyping of the spleen, by quantifying NK cells, B cells, CD4^+^ and CD8^+^ T cells, type 1 and type 2 conventional dendritic cells (cDC1 and cDC2), and GR1^+^ myeloid cells (Fig. S1a). The proportion of dendritic cells and myeloid cells were not affected, whereas the proportion of lymphocytes were affected. NK cell number and proportion were significantly reduced in both *Lrpprc*^−/KI^ and *Lrpprc*^−/−^ mice relative to *Lrpprc*^+/+^ mice (Fig. 2a, b). There was also a modest decrease in B cell and a slight increase in CD8^+^ T cell proportion in *Lrpprc*^−/−^ mice relative to *Lrpprc*^+/+^ mice (Fig. 2a). This increase in the proportion of CD8^+^ T cells resulted in a reduced CD4^+^/CD8^+^ T cell ratio in *Lrpprc*^−/−^ mice (Fig. 2a, S2a). Mice expressing at least one wild type *Lrpprc* allele (*Lrpprc*^+/−^ and *Lrpprc*^+/KI^) did not show any changes relative to *Lrpprc*^+/+^ mice (Fig. 2a, b). Taken together these results show that *Lrpprc* impacts immune cell composition in the spleen.

### Lrpprc *affects mitochondrial mass and function*

**Fig. 3:**
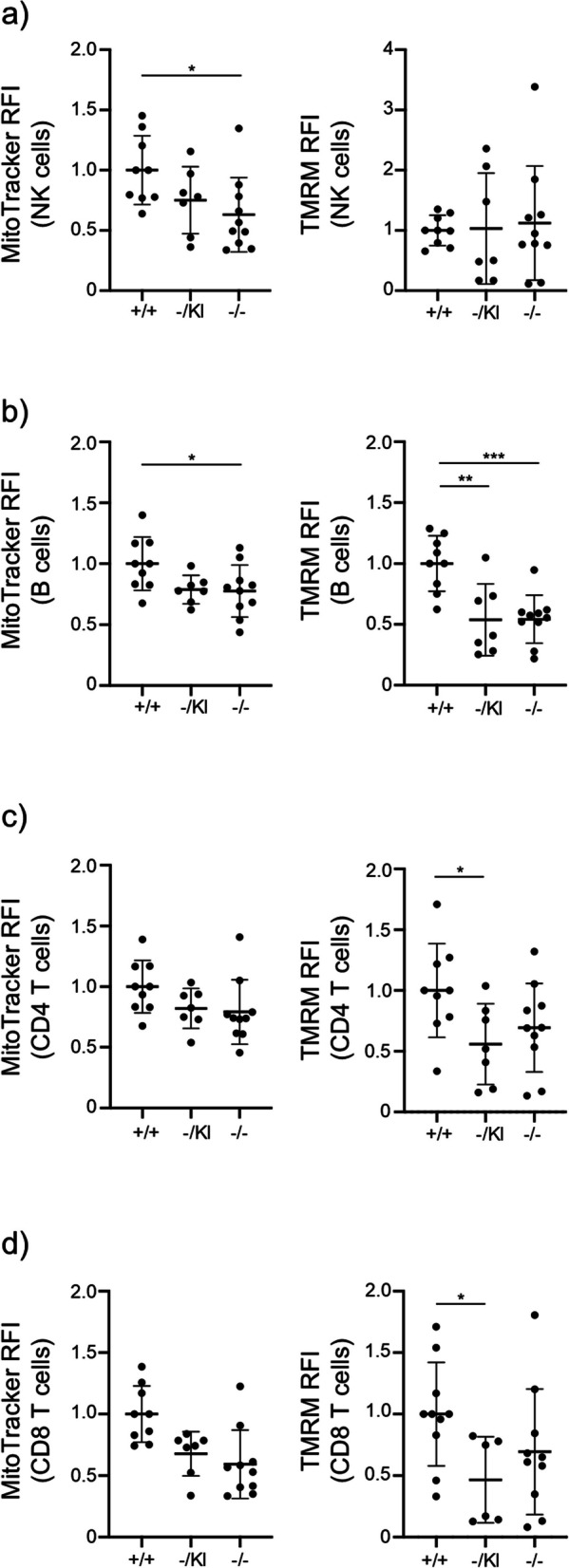
*Lrpprc* affects mitochondrial mass and mitochondrial membrane potential in immune cells. Left, compilation of MitoTracker MFI expression and, right, compilation of TMRM MFI expression for **a** NK, **b** B, **c** CD4 T, and **d** CD8 T cells from each mouse (*n* = 7–10). The data were acquired from at least three independent experiments. Each dot represents data from an individual mouse, normalized to the mean expression level in wild type mice from the same experiment. The dash depicts the mean with the standard deviation. *P < 0.05; **P < 0.01; *** *P* < 0.001. The post-gavage *Lrpprc* genotypes are indicated on the x axes

*Lrpprc* is involved in mitochondrial mRNA stability and plays an important role in mitochondrial homeostasis [14, 15, 21–23]. We assessed mitochondrial parameters, namely mitochondrial mass and mitochondrial membrane potential, in lymphocytes from the spleen of our different mouse models. NK cells, for which the number is most reduced in the spleen of *Lrpprc*^−/−^ mice, displayed reduced mitochondrial mass in *Lrpprc*^−/−^ mice (Fig. 3a). A tendency for a decreased mitochondrial mass was also observed in *Lrpprc*^−/KI^ mice, but it did not reach statistical significance (Fig. 3a). In contrast, NK cell membrane potential was comparable in mice carrying *Lrpprc*^+/+^, *Lrpprc*^−/KI^, and *Lrpprc*^−/−^ genotypes (Fig. 3a). As for NK cells, B cells from *Lrpprc*^−/−^ mice displayed a reduction in mitochondrial mass relative to *Lrpprc*^+/+^ mice (Fig. 3b). However, unlike NK cells, a decrease in membrane potential was observed for B cells in both *Lrpprc*^−/−^ and *Lrpprc*^−/KI^ mice relative to *Lrpprc*^+/+^ mice (Fig. 3b). CD4^+^ and CD8^+^ T cells from both *Lrpprc*^−/−^ and *Lrpprc*^−/KI^ mice showed a non-significant trend for reduced mitochondrial mass (Fig. 3c, d). Membrane potential was reduced in T cells from *Lrpprc*^−/KI^ and *Lrpprc*^−/−^ mice, with a significant reduction in *Lrpprc*^−/KI^ mice (Fig. 3c, d). Overall, these data show that *Lrpprc* affects mitochondrial phenotypes in immune cells.

### Lrpprc *enables cell proliferation *in vivo

**Fig. 4:**
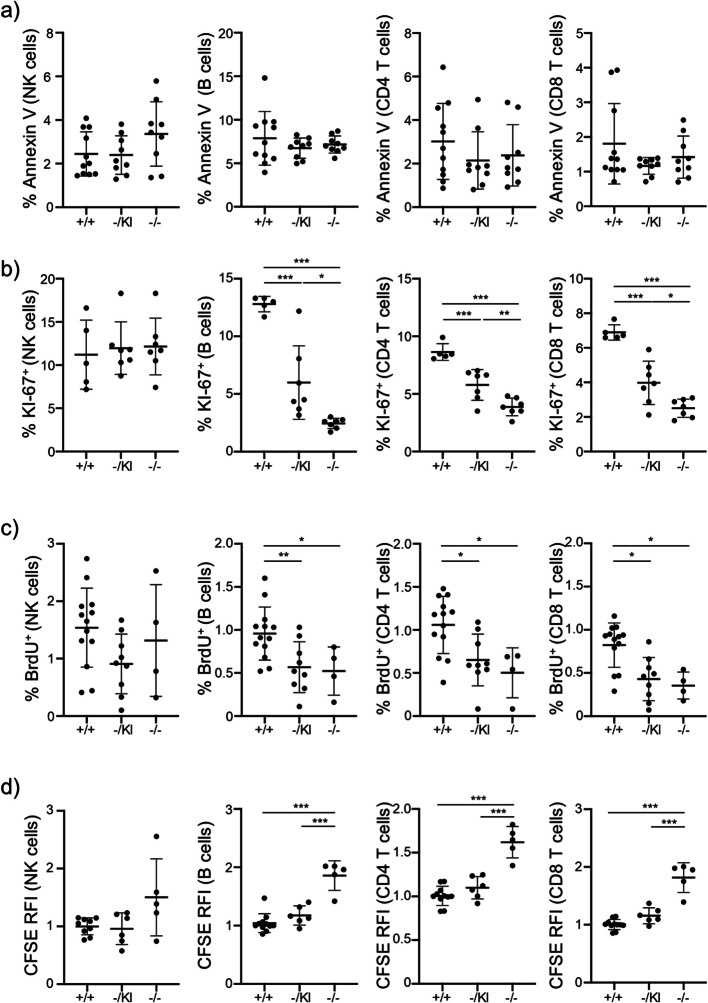
*Lrpprc* promotes cell proliferation. **a** Compilation of the percentage of apoptotic (Annexin V^+^ Viability Dye^−^) NK, B, CD4, and CD8 T cells. (*n* = 9–11). **b** Compilation of the percentage of proliferating (KI-67^+^) NK, B, CD4, and CD8 T cells. (*n* = 5–7). **c** Compilation of the percentage of proliferating (BrdU^+^) NK, B, CD4, and CD8 T cells. (*n* = 4–13). **d** Compilation of CFSE RFI expression on NK, B, CD4, and CD8 T cells (*n* = 4–11). The data were acquired from at least three independent experiments. Each dot represents data from an individual mouse, normalized to the mean expression level in *Lrpprc*^+/+^ mice from the same experiment. The dash depicts the mean with the standard deviation. *P < 0.05; **P < 0.01; *** *P* < 0.001. The post-gavage *Lrpprc* genotypes are indicated on the x axes

Mitochondria play key roles in cellular apoptosis and proliferation [27–29]. We therefore investigated if these two parameters could be at the origin of the decreased proportion of NK and B cells and the increased proportion of CD8^+^ T cells in *Lrpprc*^−/−^ relative to controls. First, we quantified the level of apoptosis and observed that *Lrpprc* did not affect apoptosis levels in all of the immune cell populations studied (Fig. 4a). Next, we quantified immune cell proliferation. In *Lrpprc*^−/−^ and *Lrpprc*^−/KI^ mice, a marked reduction of proliferation was noted for B cells, CD4^+^ and CD8^+^ T cells with both KI-67 and BrdU (Fig. 4b, c). In contrast, NK cell proliferation was unaffected (Fig. 4b, c). We confirmed these results in vitro using CFSE-labelled NK, B and T cells, stimulated with IL-15, LPS, and antibodies to CD3 and CD28, respectively. While NK cell proliferation was not significantly impaired, B cells, CD4^+^ and CD8^+^ T cells from *Lrpprc*^−/−^ mice show a clear decrease in proliferation (Fig. 4d, S3a). Together, this data show that *Lrpprc* enables cell proliferation in a cell-type specific manner, with a strong impact on B and T cells.

### Lrpprc* is not essential for hematopoietic precursors*

**Fig. 5:**
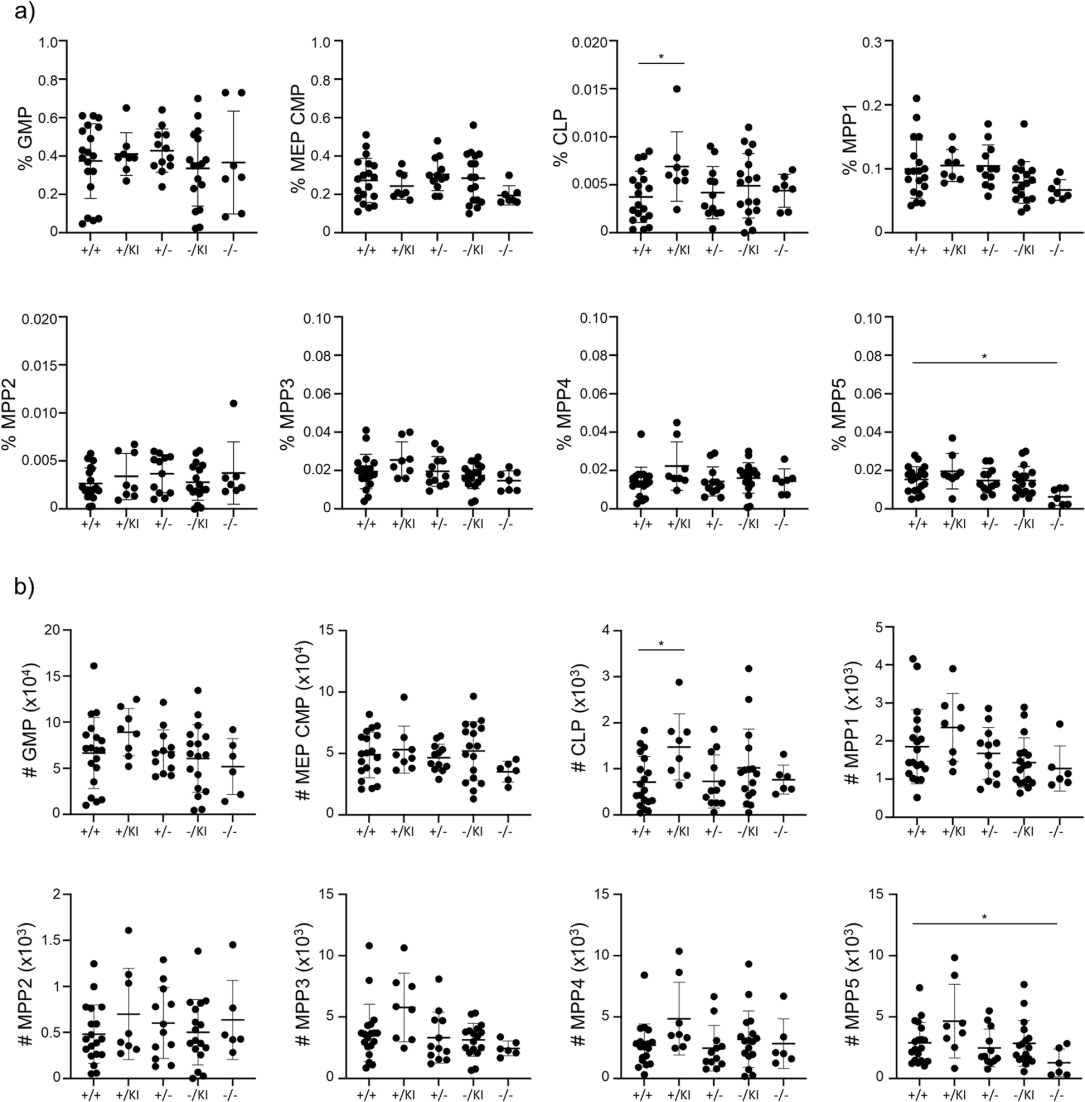
Hematopoietic precursors pool is not affected by *Lrpprc* deletion. **a** Compilation of the percentage (*n* = 7–19) and **b** absolute number (*n* = 6–19) of hematopoietic precursors gated as detailed in supplementary Fig. 1b. The data were acquired from at least three independent experiments. Each dot represents data from an individual mouse, and the dash depicts the mean with the standard deviation. *P < 0.05. The post-gavage *Lrpprc* genotypes are indicated on the x axes

**Fig. 6:**
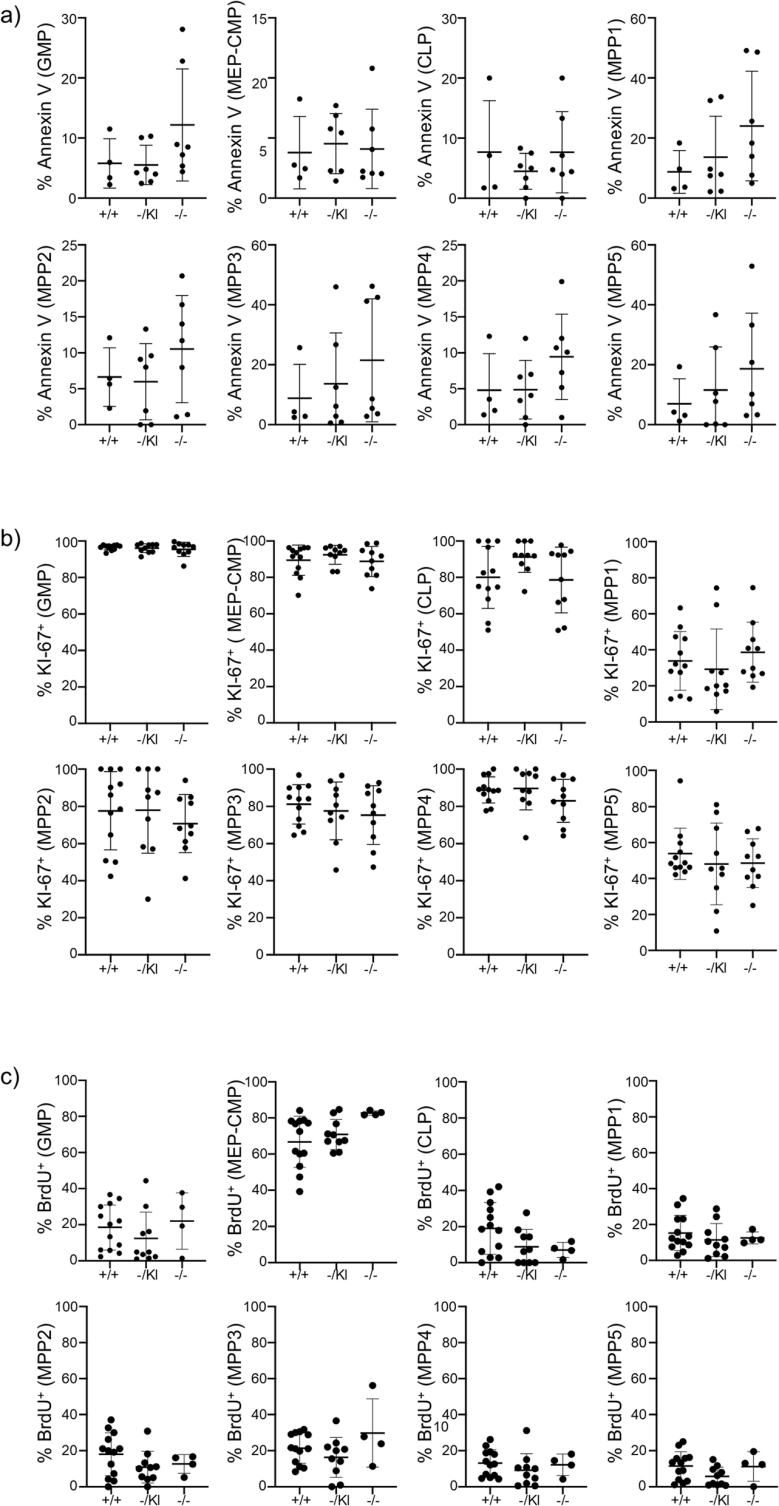
*Lrpprc* did not promote cell proliferation and apoptosis of hematopoietic precursors. **a** Compilation of the percentage of apoptotic (Annexin V^+^ Viability Dye^−^) hematopoietic precursors (*n* = 4–7). **b** Compilation of the percentage of proliferating (KI-67^+^) hematopoietic precursors (*n* = 10–12). **c** Compilation of the percentage of proliferating (BrdU^+^) hematopoietic precursors (*n* = 4–13). The data were acquired from at least three independent experiments. Each dot represents data from an individual mouse, and the dash depicts the mean with the standard deviation. The post-gavage *Lrpprc* genotypes are indicated on the x axes

We established that *Lrpprc* impacts immune cell proliferation in a cell-type specific manner. As hematopoietic stem cells are highly proliferative and responsible for establishing the pool of mature immune cells, we investigated whether immune cell precursors were affected in the bone marrow of our mouse models (Fig. S1b, S4). Specifically, among bone marrow hematopoietic cells, we quantified the proportion of granulocyte–macrophage progenitor (GMP), megakaryocyte-erythroid progenitor (MEP), common lymphoid progenitor (CLP), as well as multipotent progenitor (MPP)1 through 5 [30]. Proportions of the various precursor populations were roughly similar between *Lrpprc*^+/+^, *Lrpprc*^−/KI^, and *Lrpprc*^−/−^ mice (Fig. 5). Only MMP5 were decreased in *Lrpprc*^−/−^ mice (Fig. 5). In addition, all hematopoietic precursor populations showed similar level of apoptosis (Fig. 6a) and proliferation, measured by KI-67 expression (Fig. 6b) and BrdU incorporation (Fig. 6c). These results indicate that hematopoietic precursor numbers, apoptosis, and proliferation are not significantly affected by *Lrpprc*.

### Lrpprc *is required for B cell development*

**Fig. 7:**
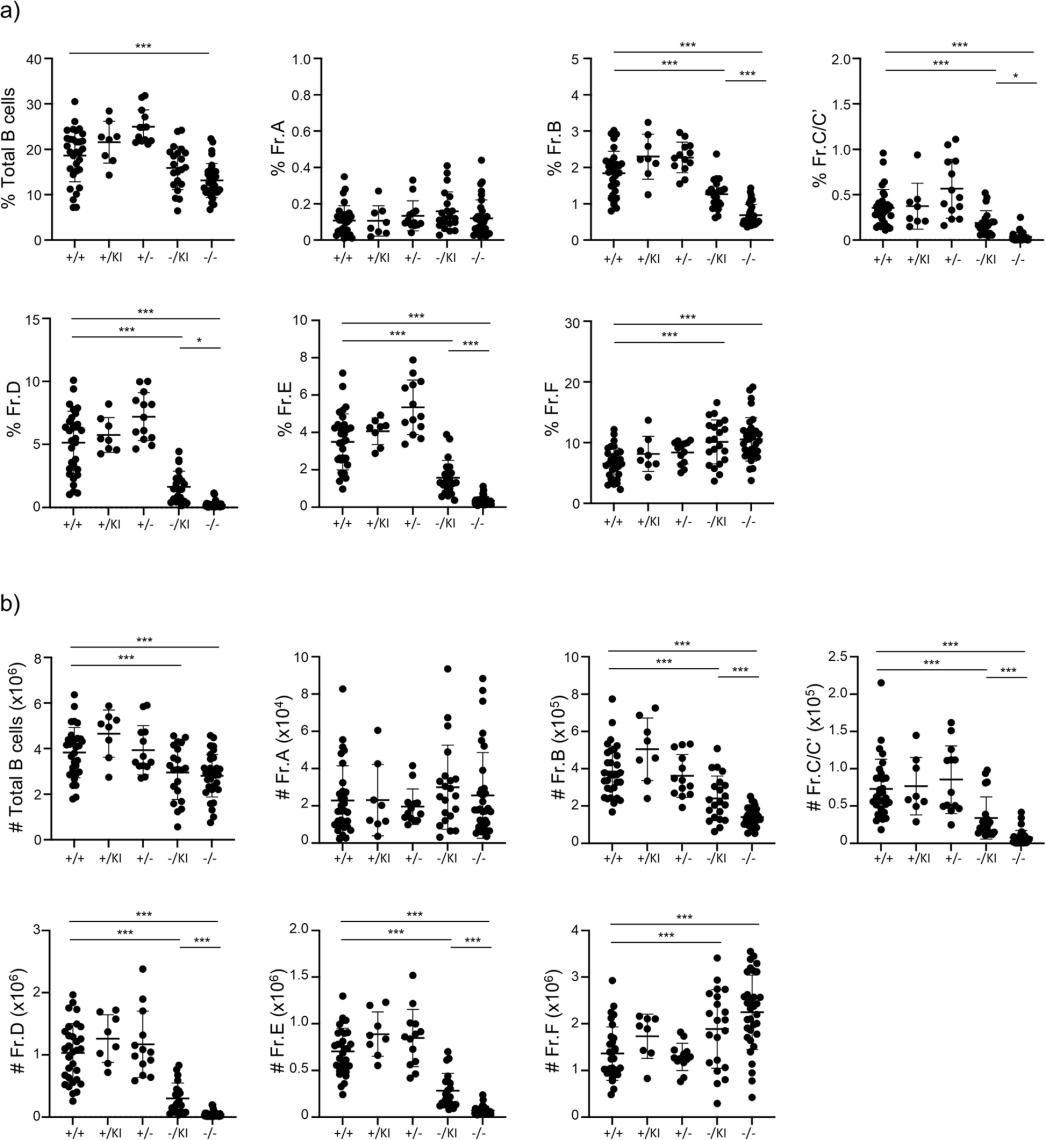
*Lrpprc* disruption impacts the B cell development. **a** Compilation of the percentage (*n* = 8–33) and **b** absolute number (*n* = 8–33) of B cell precursors cell gated as detailed in supplementary Fig. 1c. The data were acquired from at least three independent experiments. Each dot represents data from an individual mouse, and the dash depicts the mean with the standard deviation. *P < 0.05; *** *P* < 0.001. The post-gavage *Lrpprc* genotypes are indicated on the x axes

While early hematopoietic precursors did not seem to be affected by *Lrpprc* deletion, mature B cell number and proliferation potential are significantly reduced in *Lrpprc*^−/−^ mice (Fig. 2 and 4). We therefore took a closer look at B cell differentiation in the bone marrow. Total B cell numbers were significantly decreased in the bone marrow of both *Lrpprc*^−/−^ and *Lrpprc*^−/KI^ mice relative to *Lrpprc*^+/+^ mice (Fig. 7a, b). To define the B cell development stages affected by *Lrpprc*, we used Hardy’s classification [31, 32], defining B cell precursor populations by Fractions (Fr.) A through F (Fig. S1C). The proportion and number of Fr. B, C, D, and E were decreased in both *Lrpprc*^−/−^ and *Lrpprc*^−/KI^ mice relative to *Lrpprc*^+/+^ mice, with a more striking phenotype in *Lrpprc*^−/−^ mice than in *Lrpprc*^−/KI^ (Fig. 7a, b). Conversely, proportion and number of Fr. F were increased in both mice, whereas Fr. A was comparable for all strains. The decrease in B cell precursors in the bone marrow of *Lrpprc*^−/−^ and *Lrpprc*^−/KI^ mice, and the relative accumulation of mature B cells, point to a role for *Lrpprc* in B cell differentiation.

### *B cell precursor proliferation and mitochondrial properties are affected following* Lrpprc *disruption*

**Fig. 8:**
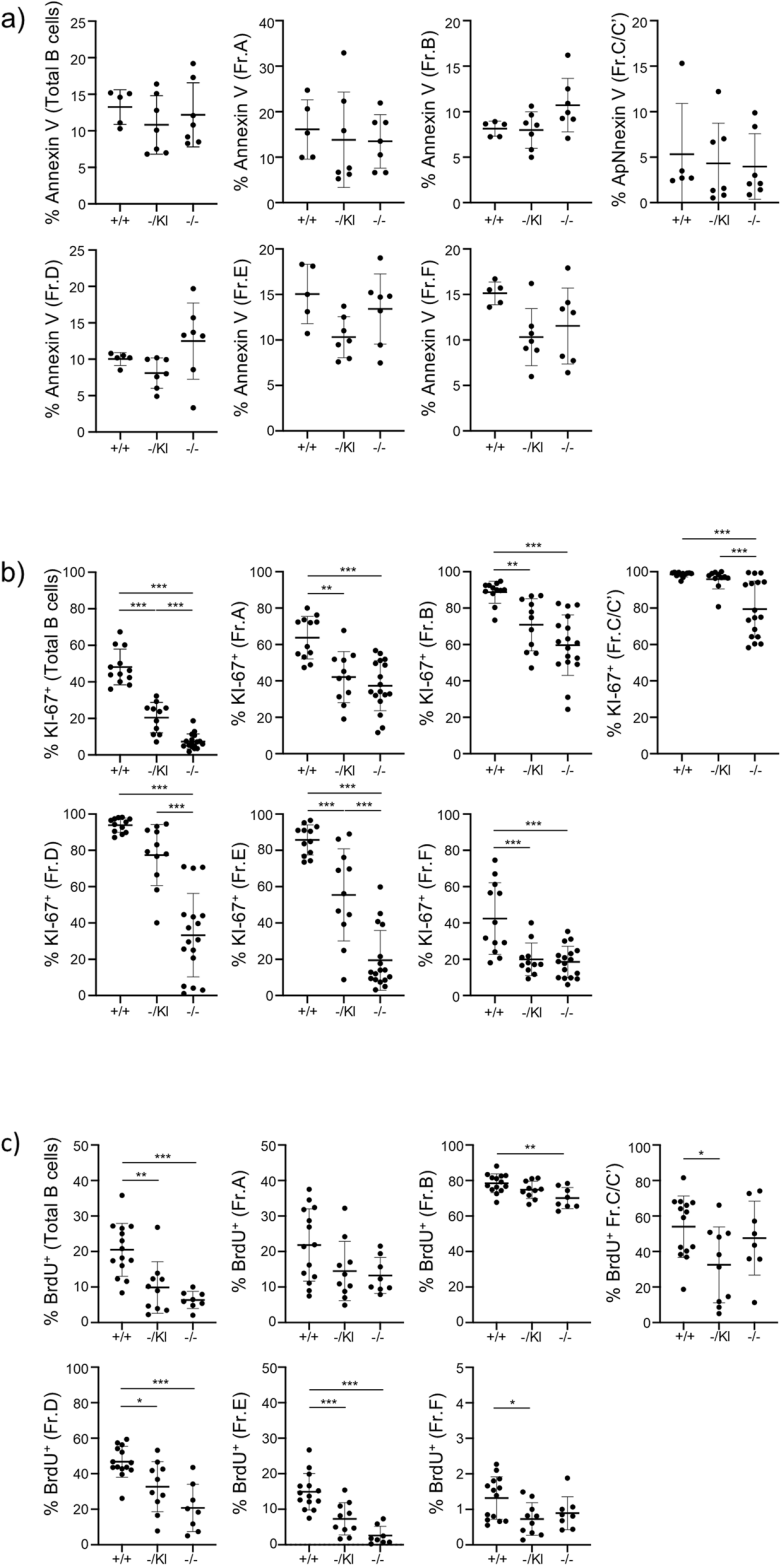
*Lrpprc* promotes the proliferation of B cell precursors. **a** Compilation of the percentage of apoptotic (Annexin V^+^ Viability Dye^−^) B cell precursors (*n* = 5–7). **b** Compilation of the percentage of proliferating (KI-67^+^) B cell precursors (*n* = 11–17). **c** Compilation of the percentage of proliferating (BrdU^+^) B cell precursors (*n* = 8–14). The data were acquired from at least three independent experiments. Each dot represents data from an individual mouse, and the dash depicts the mean with the standard deviation. *P < 0.05; **P < 0.01; *** *P* < 0.001. The post-gavage *Lrpprc* genotypes are indicated on the x axes

**Fig. 9:**
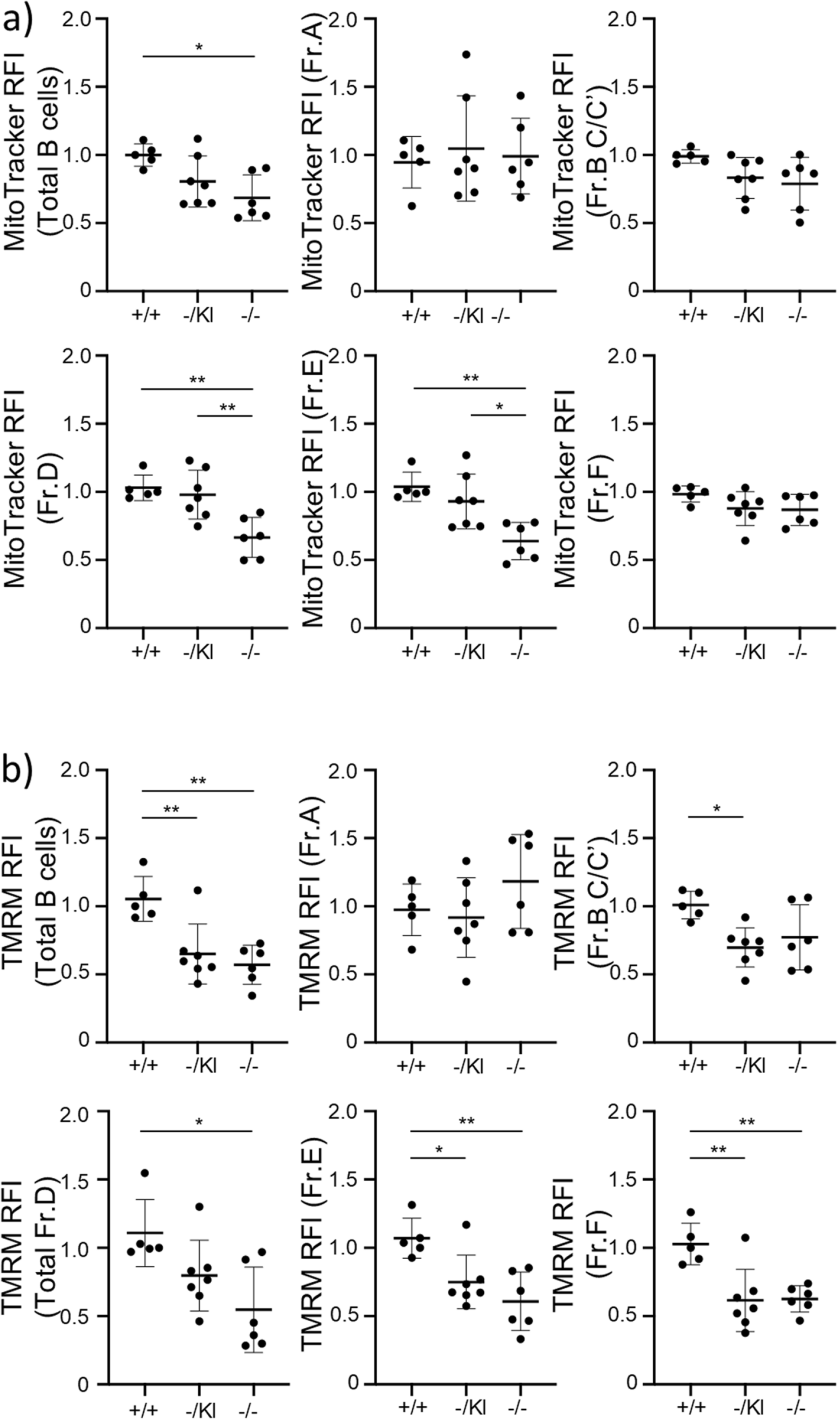
*Lrpprc* affects mitochondrial mass and mitochondrial membrane potential in B cell precursors. **a** Compilation of MitoTracker MFI expression for B cell precursors from each mouse strain (*n* = 5–7). **b** Compilation of TMRM MFI expression of B cell precursor from each mouse strain (*n* = 5–7). The data were acquired from at least three independent experiments. Each dot represents data from an individual mouse, normalized to the mean expression level in wild type mice from the same experiment. The dash depicts the mean with the standard deviation. *P < 0.05; **P < 0.01. The post-gavage *Lrpprc* genotypes are indicated on the x axes

To better understand the cause of the impaired B cell differentiation, we measured the apoptosis and proliferation rate of the different precursor populations. As for hematopoietic precursors, apoptosis was not affected in any fraction (Fig. 8a). However, proliferation was strikingly reduced throughout B cell differentiation in both *Lrpprc*^−/−^ and *Lrpprc*^−/KI^ mice, relative to *Lrpprc*^+/+^ mice. We observed a strong decrease of KI-67 expression in all stages of B cell development from *Lrpprc*^−/−^ mice, with an intermediate phenotype in *Lrpprc*^−/KI^ mice (Fig. 8b). Similar results were observed with BrdU incorporation (Fig. 8c). Next, we investigated whether, as for splenic B cells, mitochondria were impaired by *Lrpprc* disruption in B cell precursors. A decrease in mitochondrial mass was observed for total B cells and Fr.D and E in *Lrpprc*^−/−^ mice relative to *Lrpprc*^+/+^ mice (Fig. 9a). Membrane potential was also decreased in total B cells, Fr.B-C/C’, D, E and F in *Lrpprc*^−/−^ and *Lrpprc*^−/KI^ mice, relative to *Lrpprc*^+/+^ mice (Fig. 9b). These results demonstrate that *Lrpprc* strongly impacts the proliferation and mitochondrial function of B cell precursors.

## Discussion

LSFC is an orphan inherited mitochondrial disease altering cellular metabolism. We have previously demonstrated that LSFC patients show impaired response to the MMR vaccine [25]. To investigate the mechanism leading to this impaired humoral response, we generated murine models of LSFC. We initially generated *Lrpprc*^Ala354Val^ homozygous mice, that carry the orthologous human *LRPPRC* pathogenic variant described in people living with LSFC. Unfortunately, similar to *Lrpprc* total body knock-out mice, *Lrpprc*^Ala354Val/Ala354Val^ mice display an embryonic lethal phenotype. To overcome this limitation, we developed two conditional deletion models, allowing *Lrpprc* gene deletion in adult mice. The first model leads to a full body knock-out (*Lrpprc*^−/−^), whereas the second model allows for the expression of one Ala354Val mutated allele (*Lrpprc*^−/KI^). The human Ala354Val variant results in a decrease of LRPPRC protein expression [20]. Accordingly, we observed a modest decrease of Lrpprc protein levels in *Lrpprc*^+/KI^ mice, and a significant decrease in Lrpprc expression in *Lrpprc*^−/KI^ and *Lrpprc*^−/−^ mice, both showing a ~ 75% reduction in expression. Still, *Lrpprc*^−/−^ mice exhibit a more severe phenotype than *Lrpprc*^−/KI^ mice in terms of weight loss and lactate levels, suggesting that the KI allele exhibits residual activity that is not present in the *Lrpprc*^−/−^ mice. This suggests that the residual Lrpprc activity in *Lrpprc*^−/KI^ mice is sufficient to regulate blood lactate levels, at least at steady state.

*Lrpprc* appears to play cell type-specific roles in the immune system. Indeed, while *Lrpprc* disruption results in a reduced lymphocyte abundance in the spleen, the proportion and number of dendritic cells and myeloid cells appear unaffected. The impact of *Lrpprc* on mitochondria is also cell type specific. Indeed, while NK cells display a clear reduction of mitochondrial mass in *Lrpprc*^−/−^ mice, a change in mitochondrial membrane potential was mostly apparent in B cells. Finally, the T cell mitochondrial mass from *Lrpprc*^−/−^ mice trended towards a decrease relative to control mice, while a significant decrease in T cell mitochondrial membrane potential was observed in *Lrpprc*^−/KI^ mice. The cell type-specific impact of *Lrpprc* is reminiscent of a previous report demonstrating organ specific impact of *LRPPRC* variants in people living with LSFC [4]. In this study, they find that the level of cytochrome oxidase activity is tissue specific, with very low activity in the brain and liver and near normal activity in the heart and kidneys. Further studies are needed to address these cell and tissue specific differences in phenotype.

LRPPRC has an important role in mitochondria homeostasis [20, 26]. As mitochondria are central in regulating apoptosis and proliferation, we measured these parameters in immune cells. Once again, we observed cell-specific impacts of *Lrpprc*. In all cell subsets examined, apoptosis was not impacted by *Lrpprc* disruption. In contrast, B and T cells from *Lrpprc*^−/−^ mice, but not NK cells, showed a striking reduction of proliferation in vivo. As B cells are the main effectors in the humoral response to vaccines, a reduction of B cell proliferation or function may explain the impaired antibody response observed in LSFC patients [25].

NK, B, and T cells share a common progenitor in the hematopoietic lineage. However, apart from the modest decrease in the proportion of MPP5, the proportion and number of early hematopoietic precursors are not affected in *Lrpprc*^−/KI^ and *Lrpprc*^−/−^ mice. Thus, the reduction in NK cell number in the spleen cannot be attributed to a decrease in hematopoietic precursors. Because of the defective humoral response in LSFC patients, we decided to take a closer look at the development of B cells in the bone marrow. We observed an important defect in B cell development in *Lrpprc*^−/KI^ and *Lrpprc*^−/−^ mice, as evidenced by a decrease of B cell precursors from the ProB stage (Fr. B), PreB stage (Fr. C/C’ and D) and immature stage (Fr. E). Surprisingly, this impairment in B cell development did not result in severe B cell depletion in the periphery, where proportion and number of splenic B cells were only modestly decreased in *Lrpprc*^−/−^ mice. The absence of mature B cell depletion in the spleen may be explained by the model used. The conditional deletion is induced in adult mice that already possess a full pool of mature B cells with a half-life of five to six weeks [33]. It would therefore be necessary to wait more than six weeks after *Lrpprc* deletion to observe an effect on these cells, but this is impossible as the mice reach critical weight loss at this timepoint. The accumulation of mature B cells in the bone marrow may be explained by the sharp reduction in proliferation of immature fractions, which have a shorter lifespan than mature B lymphocytes [33]. The immature populations therefore disappear before the mature B lymphocytes, which consequently appear to be accumulating in the bone marrow. Altogether, our data provide strong evidence that *Lrpprc* severely impairs B cell differentiation and proliferation.

Our study reveals that *Lrpprc* contributes to B cell development, adding a novel dimension to its established immunomodulatory functions. Beyond immune cell differentiation, *LRPPRC* is also implicated in the regulation of immune responses. For instance, overexpression in hepatocellular carcinoma is associated with impaired T‐cell infiltration and immune evasion [34]. Elevated LRPPRC expression has also been documented in several malignancies, including gastric, prostate and lung cancers [35, 36], where it correlates with adverse clinical outcomes and is thought to contribute to cancer predisposition and supports its role as a critical factor in tumor progression in gastric cancer [37]. In agreement with our findings, these studies suggest that LRPPRC regulates cell proliferation.

A limitation of our current study is that we investigate the impact of systemic deletion of *Lrpprc*. This systemic deletion causes an increase in lactate levels in *Lrpprc*^*−/−*^ mice, and a decrease in body weight in both *Lrpprc*^−/KI^ and *Lrpprc*^−/−^ mice. This suggests that the global health status of the mice is affected. As such, it may indirectly impact on the immune system. Of interest, a recent study by Pioli et al*.* demonstrated that lymphopoiesis is attenuated upon hepatocyte-specific deletion of the cytochrome c oxidase assembly factor Sco1 [38]. Most of the lymphoid phenotypes in *Lrpprc*^−/KI^ and *Lrpprc*^−/−^ mice phenocopy the hepatocyte-specific Sco1 deletion. This suggests that liver metabolites might be driving at least some of the immune phenotypes observed in the *Lrpprc*^−/KI^ and *Lrpprc*^−/−^ mice. Additional studies with immune cell-specific conditional deletion of *Lrpprc* will help define the intrinsic and extrinsic roles of *Lrpprc* in the immune system.

Overall, this study demonstrates the cell-specific impact of deletion or variant of *Lrpprc* on the immune system in the steady state. *Lrpprc* plays a crucial role in the proliferation of lymphocyte subsets in the periphery and the bone marrow. Future studies are needed to understand how these phenotypes impact the immune response to different stimuli, to better understand the origin of the failed vaccine response in LSFC patients.

## Materials and methods

### Mice

The GT Rosa ERT2 B6.129-Gt(ROSA)26Sor^*tm1(cre/ERT2)Tyj*^/J strain (#008463) was purchased from The Jackson Laboratory. This strain ubiquitously expresses a Cre-recombinase linked to human estrogen. *Lrpprc* lox mice (*Lrpprc*^fl/fl^) has been previously described [26]. *Lrpprc*^+/KI^ was generated by Ozgene Pty Ltd. in Australia. They utilized homologous recombination with traditional gene targeting techniques to create a knock-in (KI) mouse model on a C57BL/6 J background. This mouse model contains the Ala354Val variant in exon 9 of the mouse *Lrpprc* gene. Confirmation of the variant was achieved through Whole Genome Sequencing of genomic DNA from the tails of heterozygous animals. *Lrpprc*^−/−^ mice were obtained by breeding *Lrpprc*^fl/fl^ mice, which bear *lox* sites in intron 3 and 5 of *Lrpprc *[26], with GT-Rosa CreERT2^+/−^ to obtain *Lrpprc*^fl/fl^.GT-Rosa CreERT2^+/−^ mice. The *Lrpprc*^−/KI^ mice were obtained by breeding *Lrpprc*^fl/fl^.GT-Rosa CreERT2^+/−^ mice with *Lrpprc*^+/KI^ to obtain *Lrpprc*^fl/KI^.GT-Rosa CreERT2^+/−^ mice. We also generated heterozygous mice, namely *Lrpprc*^+/KI^ and GT-Rosa^cre/ERT2+/−^.*Lrpprc*^+/fl^ (*Lrpprc*^+/−^). The data from *Lrpprc*^+/+^ mice carrying the following genotypes were pooled and used as littermate control; Cre^−/−^-*Lrpprc*^fl/fl^, Cre^−/−^-*Lrpprc*^+/fl^, Cre^−/−^-*Lrpprc*^+/+^ or Cre^−/+^-*Lrpprc*^+/+^; expectedly, no differences in phenotypes were observed among these different genotypes as they all expressed two functional alleles of *Lrpprc*. The genotype of all transgenic mice was verified by PCR. Mice used in the experiments were born, housed and raised in a single room of the pathogen-free animal facility at Maisonneuve-Rosemont Hospital. To induce *Lrpprc* deletion, six- to twelve-week-old mice were gavaged twice at 1-day intervals with 200uL of tamoxifen 50 mg/mL (Sigma, St Louis, MO, USA). A period of six weeks was allowed after gavage. For phenotyping experiments, male and female mice were used, with no significant difference in phenotype observed when data were separated by sex, data from both sexes were therefore pooled. The Maisonneuve-Rosemont Hospital Ethics Committee, supervised by the Canadian Council on Animal Care, approved the breeding of the mice and the experimental procedures (protocol 2020–1480).

### Immunoblotting

Proteins from snap-frozen spleen tissues were extracted using a Tris–HCl lysis buffer supplemented with anti-protease and anti-phosphatase agents. Each sample (40 μg of protein) was loaded into a 12.5% polyacrylamide gel. Following migration, the proteins were transferred to a 0.2 µm nitrocellulose membrane and used for the detection of *Lrpprc* (1:1000; LRP130 Rabbit Ab, cat. PA5-22,034, ThermoFisher Scientific). β-actin antibody (1:50 000; β-actin HRP (C4), cat. sc-47778, Santa Cruz Biotechnologies) was used for normalization. The Precision Plus Protein Dual Color Standards (Bio-Rad, #1,610,374) were used as a size ladder. Protein levels were assessed using a ChemiDoc Imaging System (Biorad).

### pH and lactate blood levels

Blood was collected six weeks after gavage. pH and lactate were quantified on the hospital biochemistry platform using Instrumentation Laboratory GEM5000 analyzers, within 30 min of blood draw.

### Flow cytometry

Organs were passed through a 70 μm cell strainer (BD Biosciences; Franklin Lakes, NJ, USA) to yield single-cell suspensions. Red blood cells were lysed with NH_4_Cl solution. Cell suspensions were labeled with combinations of the antibodies targeting the following proteins, with the clone names in parenthesis: CD3 (145-2C11 or 17A2), CD8α (53–6.7), CD19 (6D5), NKP46 (29A1.4), CD11b (M1/70), GR1 (RB6-8 C5), IA-IE (M5/114.15.2), CD11c (N418), B220 (RA3-6B2), IgM (RMM1), IgD (11-26c.2a), CD24 (M1/69), CD43 (S11), BP-1 (6C3), TER-119 (TER-119), CD117 (2B8), CD150 (TC15-12F12.2), CD16/32 (93), CD48 (HM48-1), CD135 (A3F10), CD127 (A7R34), TCRβ (H57-597), CD44 (IM7), CD49b (DX5), CD122 (TM-B1) purchased from BioLegend (San Diego, CA, USA); CD4 (RM5-5), SCA-1 (D7), CD8α (53–6.7), KI-67 (B56) purchased from BD Biosciences. MitoTracker Green and Tetramethylrhodamine, methyl ester (TMRM) were used according to the manufacturer’s instructions. Foxp3/Transcription Factor Staining Buffer Set (eBioscience) was used for KI-67 staining. Dead cells were stained using the LIVE/DEAD Fixable Zombie Aqua Dye, purchased from (ThermoFisher Scientific, Waltham, MA, USA). Annexin-V (BioLegend) in its appropriate buffer (10 mmol/L Hepes, 150 mmol/L NaCl, 5 mmol/L KCl, 1 mmol/L MgCl_2_, 1,8 mmol/L CaCl_2_) was added for 10 min without washing, prior to flow cytometry data acquisition. All samples were acquired using LSRFortessa™ X-20 (BD Biosciences) and were analyzed using the FlowJo software (BD Biosciences).

### BrdU

Mice were injected intraperitoneally with 20 mg of BrdU (5-Bromo-2’-deoxy-uridine, Sigma) 24 h before analysis. Cells are first stained for extracellular markers, prior to permeabilization with the BD Bioscience Fixation and Permeabilization kit for 30 min at 4 °C. Cells are washed and permeabilized again for 5 min with the same kit. Cells are then treated with DNAse I (Sigma) for 1 h at 37 °C. After washing, cells are stained with an antibody against BrdU (BD Biosciences) for 30 min 4 °C, prior to flow cytometry data acquisition.

### CFSE staining

Total spleen cells were labelled with CFSE (2 μM) (Invitrogen) and washed twice in cold supplemented RPMI before culture. CFSE-labelled cells were cultured 48 h in the presence of LPS (1 μg/mL, Sigma) for B cells, anti-CD3 and anti-CD28 (2 μg/ml) for T cells, and 72 h with IL-15 (100 ng/ml) for NK cells, compared to compete RPMI medium (RPMI supplemented with 10% fetal bovine serum 1% penicillin–streptomycin, 1% HEPES and 0.1% 2-mercaptoethanol) in 96 well plate (flat, SARSTEDT, Nümbrecht, Germany). CFSE dilution was monitored by flow cytometry. Data is presented as CFSE RFI (relative fluorescence intensity) of stimulated cells, using cells from *Lrpprc*^+/+^ mice cells as controls.

### Statistical analysis

Data were tested for significance using a one-way ANOVA. Numbers of animals used per group (n) are indicated in the Figure legends. The minimal significance threshold was set at 0.05 for all tests.

## Supplementary Information

Supplementary Material 1.

## Data Availability

All data are included in the manuscript. Access to raw data, such as flow cytometry data, are available from the authors upon reasonable request.
