# Supplementary material for: Disruption of Lrpprc affects B cell development and proliferation in a mouse model of Leigh Syndrome French Canadian type
Source: J Rare Dis (Berlin). 2025 Jul 1;4(1):31. doi: 10.1007/s44162-025-00094-x (PMC12209026; doi:10.1007/s44162-025-00094-x)
Supplement: Supplementary file 1 — Supplementary Material 1. [file 44162_2025_94_MOESM1_ESM.pdf]

Supplementary figures

Disruption of LRPPRC affects B cell development and proliferation in a mouse model of Leigh Syndrome French Canadian type

AUTHORS

Adrien Fois[1,2], Sonia Deschênes[3], Capucine Bourel[1,2], Claudine Beauchamp[3], Félix Lombard-Vadnais[1], Matthieu Ruiz[3,4], Guy Charron[3], Lise Coderre[1,2], LSFC Consortium , John D. Rioux[3,5], Sylvie Lesage[1,2]

Figure S1

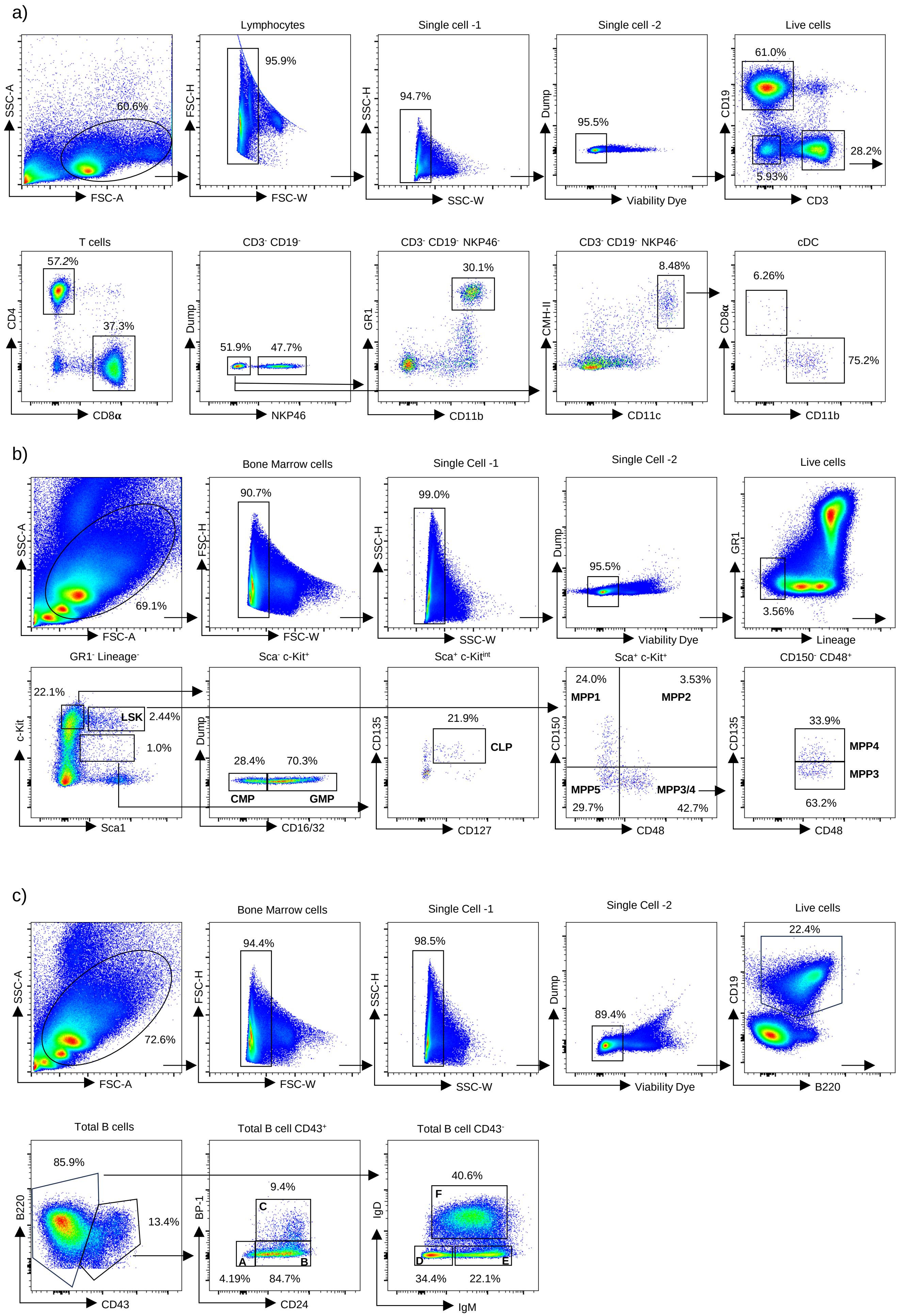
a)
SSC-A
FSC-A
60.6%
Lymphocytes
FSC-H
FSC-W
95.9%
Single cell -1
SSC-H
SSC-W
94.7%
Single cell -2
Dump
Viability Dye
95.5%
Live cells
CD19
CD3
61.0%
5.93%
28.2%
T cells
CD4
CD8α
57.2%
37.3%
CD3- CD19-
Dump
NKP46
51.9%
47.7%
CD3- CD19- NKP46-
GR1
CD11b
30.1%
CD3- CD19- NKP46-
CMH-II
CD11c
8.48%
cDC
CD8α
CD11b
6.26%
75.2%
b)
SSC-A
FSC-A
69.1%
Bone Marrow cells
FSC-H
FSC-W
90.7%
Single Cell -1
SSC-H
SSC-W
99.0%
Single Cell -2
Dump
Viability Dye
95.5%
Live cells
GR1
Lineage
3.56%
GR1- Lineage-
c-Kit
Sca1
22.1%
LSK
2.44%
1.0%
Sca- c-Kit+
Dump
CD16/32
28.4%
70.3%
CMP
GMP
Sca+ c-Kitint
CD135
CD127
21.9%
CLP
Sca+ c-Kit+
CD150
CD48
24.0%
3.53%
MPP1
MPP2
MPP5
MPP3/4
29.7%
42.7%
CD150- CD48+
CD135
CD48
33.9%
MPP4
MPP3
63.2%
c)
SSC-A
FSC-A
72.6%
Bone Marrow cells
FSC-H
FSC-W
94.4%
Single Cell -1
SSC-H
SSC-W
98.5%
Single Cell -2
Dump
Viability Dye
89.4%
Live cells
CD19
B220
22.4%
Total B cells
B220
CD43
85.9%
13.4%
Total B cell CD43+
BP-1
CD24
9.4%
C
A
B
4.19%
84.7%
Total B cell CD43-
IgD
IgM
40.6%
F
D
E
34.4%
22.1%

Supplementary Fig. 1: Gating Strategy. (a) Gating strategy for spleen cell analysis. After doublet exclusion and live gating, immune cells are selected as follows: B cells, $CD19^{+}$; CD4 T cells, $CD3^{+}CD4^{+}$; CD8 T cells, $CD3^{+}CD8^{+}$; NK cells, $CD3^{-}CD19^{-}NKP46^{+}$; granulocytes, $CD3^{-}CD19^{-}NKP46^{-}GR1^{+}CD11b^{+}$; and cDCs $CD3^{-}CD19^{-}NKP46^{-}CD11c^{+}MHCII^{+}$, with cDC1 as $CD8\alpha^{+}$ and cDC2 as $CD11b^{+}$. (b) Gating strategy for hematopoietic precursor cell analysis. After doublet exclusion, live gating, lineage (CD3, CD19, B220, CD11b, TER119) and GR1 exclusion, the hematopoietic precursors are gated as follows: GMP, $Sca1^{-}c\text{-}Kit^{+}CD16/32^{+}$; MEP-CMP, $Sca1^{-}c\text{-}Kit^{+}CD16/32^{-}$; CLP, $Sca1^{+}c\text{-}Kit^{int}CD135^{+}CD127^{+}$. MPP are gated on $Sca1^{+}c\text{-}Kit^{hi}$, then identified by: MPP1, $CD48^{-}CD150^{+}$; MPP2, $CD48^{+}CD150^{+}$; MPP3, $CD48^{+}CD150^{-}CD135^{-}$; MPP4, $CD48^{+}CD150^{-}CD135^{+}$; MPP5, $CD48^{-}CD150^{-}$. (c) Gating strategy for B cell precursor analysis. After doublet exclusion and live gating, B cells are gated on $CD19^{+}$ $B220^{+}$. Fractions A-C and D-F are separated by expression of CD43. Fractions A to C are separated among $CD43^{+}$ cells by the expression of BP1 and CD24. Fraction A, $BP1^{-}$ $CD24^{-}$; Fraction B, $BP1^{-}$ $CD24^{+}$; Fraction C, $BP1^{+}$ $CD24^{+}$; Fractions D to F are separated among $CD43^{-}$ cells by the expression of IgM and IgD. Fraction D, IgM^{-} IgD^{-}; Fraction E, IgM^{+} IgD^{-}; Fraction F, IgM^{+} IgD^{+}.

Figure S2

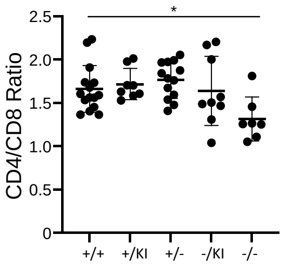

Supplementary Fig. 2: CD4/CD8 ratio. Compilation of CD4/CD8 T cell ratio in the spleen (n=7-15). The data were acquired from at least three independent experiments. Each dot represents data from an individual mouse, the dash depicts the mean with the standard deviation. *P < 0.05. The post-gavage LRPPRC genotypes are indicated on the x axis.

Figure S3

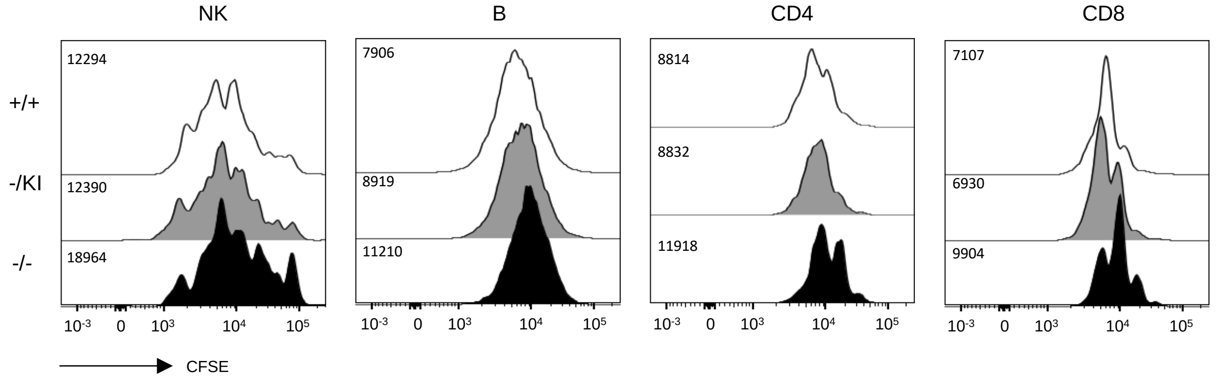

Supplementary Fig. 3: LRPPRC promotes cell proliferation in vitro. Representative histograms of CFSE dilution on NK, B, CD4 and CD8 T cells. MFIs are indicated for each mouse. The post-gavage LRPPRC genotypes are indicated.

Figure S4

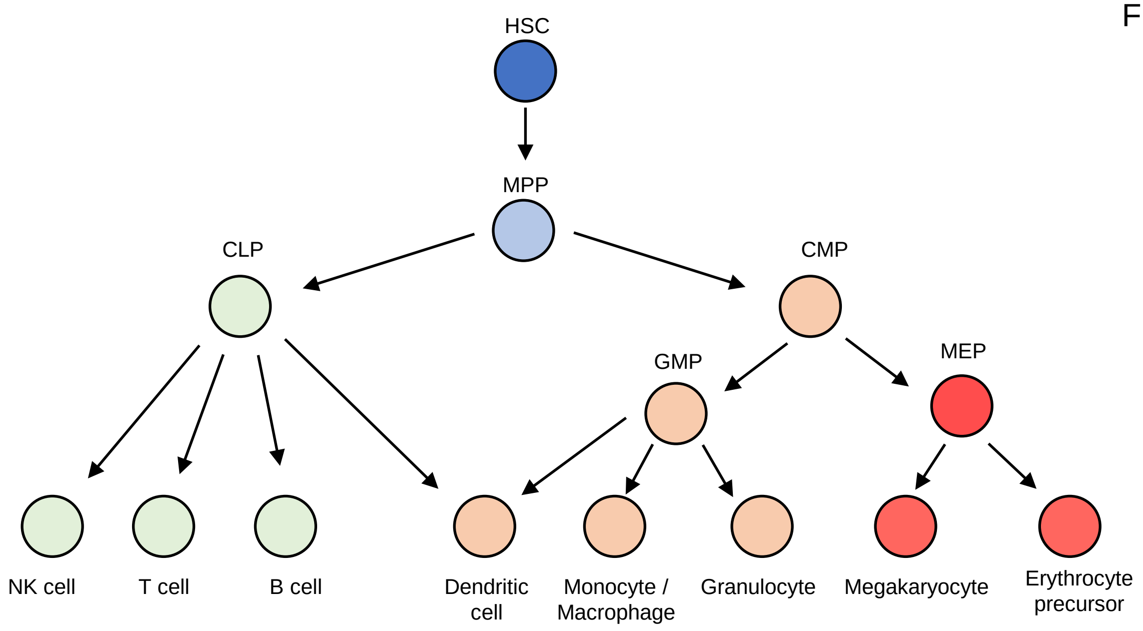

Supplementary Fig. 4: Schematic representation of hematopoietic differentiation.
